# Use of a Biomimetic Scaffold for the Treatment of Osteochondral Lesions in Early Osteoarthritis

**DOI:** 10.1155/2018/7937089

**Published:** 2018-11-01

**Authors:** Vincenzo Condello, Giuseppe Filardo, Vincenzo Madonna, Luca Andriolo, Daniele Screpis, Marco Bonomo, Marcello Zappia, Luca Dei Giudici, Claudio Zorzi

**Affiliations:** ^1^Joint Preservation and Reconstructive Surgery and Sports Medicine Unit, Humanitas Castelli Clinic, Bergamo, Italy; ^2^Applied and Translational Research (ATR) Center, IRCCS Istituto Ortopedico Rizzoli, Bologna, Italy; ^3^Clinica Ortopedica e Traumatologica 2, IRCCS Istituto Ortopedico Rizzoli, Bologna, Italy; ^4^Department of Orthopaedics, Ospedale IRCCS, Sacro Cuore–Don Calabria, Negrar, Verona, Italy; ^5^Department of Health Sciences, University of Molise, Campobasso, Italy; ^6^Varelli Institute, Naples, Italy; ^7^Orthopaedic Unit, Casa di Cura “Villa Igea” Hospital, Ancona, Italy

## Abstract

The aim of this study is to investigate clinical and radiographic outcomes of a biomimetic scaffold for the treatment of osteochondral knee lesions in patients with early OA. Study population was represented by 26 patients with a mean age of 44 years affected by early OA. Inclusion criteria were two episodes of knee pain for more than 10 days in the last year, Kellgren-Lawrence OA grade 0 or I or II, and arthroscopic findings of cartilage defects. Nineteen patients had a previous surgery, 11 of which were revision surgeries of osteochondral unit. All patients were treated with a biomimetic scaffold with a tri-layered structure of type I equine collagen and magnesium-enriched hydroxyapatite. Clinical outcomes were evaluated using the IKDC, Lysholm, VAS, KOOS, and Tegner scores at baseline and at an average follow-up of 35 months. Magnetic resonance imaging (MRI) was performed at follow-up time in 19 patients. Clinical outcomes showed significant improvement in VAS, Lysholm, IKDC subjective score, and KOOS subscales in 69% of the patients. Complication rate of this cases series was 11%, with no surgical failure, although 31% of patients did not reach a significant improvement and were thus considered as clinical failure. MRI analysis showed integration of the scaffold only in 47% of the patients, with partial regeneration of the subchondral bone. No correlation between clinics and radiological images was found. The use of a biomimetic osteochondral scaffold in the setting of an early OA, alone or associated with other procedures, appeared to be a valid and safe option, able to provide good and stable clinical outcomes with high patient's satisfaction and low complication rate.

## 1. Introduction

Osteoarthritis (OA) is one of the most common orthopaedic conditions, generally affecting patients over 50 years old, with joint pain and decreased function [1]. This disorder, chronic and degenerative in nature, presents early phases with signs and symptoms that in recent years were classified under the term “early OA” (EOA). EOA, as described by Luyten et al. [2] is characterized by at least two episodes of joint pain for more than 10 days in the last year, radiographic Kellgren-Lawrence classification up to grade 2, and arthroscopic findings of ICRS cartilage defects grade III or IV with softening and swelling of the surrounding cartilage. The main concern of this condition is often the young age of the affected patients, which represents an issue for joint metal replacement. Thus, EOA patients could benefit from a biological treatment approach to restore the articular surface and avoid or at least delay prosthetic resurfacing. In this light, the greatest challenge is represented by the unfavorable environment characterizing OA joints, which was shown to significantly decrease the potential of traditional chondral and osteochondral regenerative procedure [3, 4]. Thus, surgeons need to consider both the aim to allow a full and prompt return to expected activity and the need to treat the articular cartilage defect with an effective treatment, in the attempt to arrest, or at least delay, the progression of the disease.

OA degeneration involves the entire osteochondral unit. Thus, to address both cartilage and the subchondral bone, biphasic scaffolds have been developed and recently gained increasing credit for the treatment of osteochondral lesions. Several in vitro and in vivo experiments [5–7] showed good tissue formation even without the addition of cells: the scaffold appeared to induce in situ regeneration through cells from bone marrow, leading to the formation of cartilage-like tissues. Moreover, several clinical studies [8–13] demonstrated its feasibility, efficacy, and safety, and good results maintained from short- to medium-term follow-ups. More recently, they were proposed to restore the osteochondral unit also in more complex cases, like tibial plateau fracture [14] and osteonecrosis [15], and even in patients presenting an osteochondral defect in the setting of EOA, with promising preliminary clinical results [16]. Nevertheless, results in such challenging indication for scaffold implantation are still sparse and preliminary.

Thus, the aim of this study was to assess the safety of the procedure, and the clinical and imaging outcomes of a biphasic biomimetic scaffold for the treatment of osteochondral defects in the setting of EOA.

## 2. Materials and Methods

The study population is represented by patients affected by knee early osteoarthritis (EOA) and treated with an osteochondral scaffold implantation. The inclusion criteria for treatment, according to the criteria defined by Luyten et al. [2], were knee pain with at least two episodes of pain for more than 10 days in the last year, Kellgren-Lawrence grading less than or equal to 2 degrees, and arthroscopic findings of cartilage lesions of III or IV degree of ICRS with at least surrounding softening and swelling of the cartilage. Exclusion criteria were lesions on the tibial plateau, osteochondritis dissecans (OCD), and patients with uncorrected (not treated) lower limb malalignment >5° and instability of the knee. Patients presenting infectious, neoplastic, metabolic, and inflammatory pathologies, as well as those not able to comply with the required postoperative rehabilitation regimen, were also excluded from this study.

All patients have been treated with a biomimetic scaffold with a tri-layered structure that reproduced the osteochondral tissue (MaioRegen, Finceramica SpA, Faenza, Italy). The lower layer consists of a mineralized blend of type I equine collagen (30%) and hydroxyapatite (70%) reproducing the subchondral bone layer. The intermediate layer (tide mark-like) consists of a combination of type I collagen (60%) and hydroxyapatite (40%), whereas the superficial layer consists of type I collagen and has a smooth surface to mimic the cartilage surface.

### 2.1. Surgical Procedure

The surgical procedure was performed with the patient under general or spinal anesthesia and in the supine position with a pneumatic tourniquet around the proximal thigh. An arthroscopic joint evaluation was performed to confirm the diagnosis of EOA. The defects were exposed through a medial, mini-arthrotomic, paratendineous approach for the medial femoral condyle and trochlea lesions. In case of patellar lesion, a medial parapatellar approach was used; after capsulotomy, the patella was everted in order to visualize and to treat the osteochondral lesion; after implantation, the medial retinaculum of the patella was sutured in order to avoid postoperative patellar maltracking. Regardless of the surgical approach, the chondral defect was prepared with an osteotome and the arthroscopic shaver, by removing the sclerotic subchondral bone layer. A 7 mm deep lodging with perpendicular sides was created to allow for press-fit fixation of the implant [8]. Stability was then visually and manually tested by cyclic flexion-extension of the knee, both before and after tourniquet removal. If additional stability was required, a modified surgical technique was used, applying fibrin glue to cover the scaffold surface and the host-scaffold interface while avoiding the presence of fibrin glue between the bottom of the scaffold and subchondral bone. Fibrin glue can protect the superficial layer, which is the most susceptible to the proinflammatory factors of the joint environment. However, cases with significant synovial inflammation were considered contraindicated for surgery.

### 2.2. Post-Surgery Rehabilitation and Evaluation

Patients were hospitalized for an average of 3 days and maintained the knee in full extension with a brace for 7 days. Then patients started the rehabilitation program based on the progressive recovery of range of motion (ROM), quadriceps strength, and the weight bearing according to the associated surgery and the location of the chondral defect treated. Overall, partial weight bearing was allowed at 6 weeks for 2 weeks in cases of femorotibial lesion treated, whereas partial weight bearing was allowed at 2 weeks for 2 weeks in cases of patellofemoral lesion treated. Proprioceptive exercises began at full weight bearing recovery.

Patients were evaluated at a mean follow-up of 35 months. The clinical outcome was evaluated using the International Knee Documentation Committee score (IKDC) [17], Lysholm score [18], Visual Analogue Scale (VAS) [19], Knee injury and Osteoarthritis Outcome Score (KOOS) [20], and Tegner score for preoperatory (baseline status) and follow-up visit. Any kind of adverse event was recorded at the follow-up visit. The operation was deemed to have failed if the patient needed reoperation because of symptoms due to primary defects. For failed patients, the last clinical evaluation before reoperation was considered for final evaluation. Besides surgical failures, patients without a clinically significant improvement (10 IKDC subj points) with respect to the basal evaluation were considered clinical failures [21].

MRI evaluation was performed using a 1.5-T superconducting magnet (General Electric Co, Fairfield, Connecticut) with a dedicated quadrature detection knee coil (Quadknee; diameter, 18 cm). The following sequences were used for graft evaluation: sagittal fast spin echo, proton density weighted with fat saturation, sagittal dual fast spin echo T2 weighted and proton density weighted, and axial fast imaging employing steady-state acquisition, axial 3-dimensional gradient echo with fat suppression and axial fast spin echo, and proton density weighted with fat saturation. The MOCART scoring system [22] was applied for the evaluation of the grafts. All imaging evaluations were blindly performed by an orthopaedic surgeon and a musculoskeletal radiologist experienced in cartilage regeneration procedures. After an initial independent assessment, all images were reviewed in consensus.

### 2.3. Statistical Methods

All continuous data are expressed in terms of mean ± SD, and categorical variables are expressed as proportions or percentages. The Kolmogorov Smirnov test was performed to test normality of continuous variables. Repeated Measures GLM with post hoc Sidak correction for multiple comparisons was performed to compare normally distributed scores at the different follow-up times. The Friedman nonparametric test with Wilcoxon Test post hoc test with Holm correction for multiple comparisons was performed to compare not normally distributed scores at the different follow-up times. The ANOVA test was performed to assess the between-group differences of continuous and normally distributed and homoscedastic data; the Mann Whitney test was used otherwise. The Spearman rank Correlation was used to assess correlations between scores and continuous data. Fisher's exact test was performed to investigate the relationships between grouping variables [23]. The analysis on the MRI findings was evaluated by the Monte Carlo method for small samples. For all tests p<0.05 was considered significant.

## 3. Results

The study group consisted in 26 cases (18 males, 8 females). Mean age was 43.8 ± 11.2 years and mean BMI was 27.3 ± 4.3; 15 patients were affected on the right knee and 11 on the left knee, and the mean time since symptoms onset was 20.0 ± 14.9 months; 18 patients presented a IV degree and 8 patients a III degree of ICRS chondral lesion, respectively. The sites of the lesions were medial femoral condyle (n=17), trochlea (n=6), and patella (n=3). Etiology was rated as microtraumatic or degenerative in 19 cases and posttraumatic (not acute setting) in 7 cases. Only 9 were active amateur sport patients. Nineteen patients had at least one previous surgery, 11 of which related to chondral or osteochondral categories. In particular, 2/3 patients with patellar lesions had undergone previous surgical procedures (3 and 7 surgeries, respectively). Most of the patients (n=18) had undergone one or more associated surgical procedures: 9 tibial osteotomies and 1 femoral osteotomy, 5 patellar realignments, 2 medial meniscal transplantations, 2 ACL reconstructions, 2 synovial debridements, 1 microfracture (in another site), and 1 medial and 1 lateral meniscectomy.

**Figure 1:**
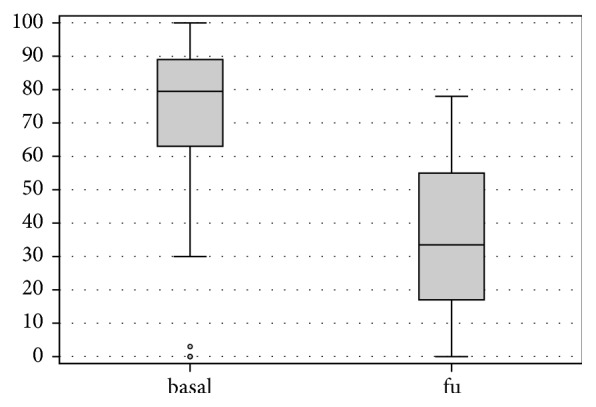
VAS score at basal level and at final follow-up.

**Figure 2:**
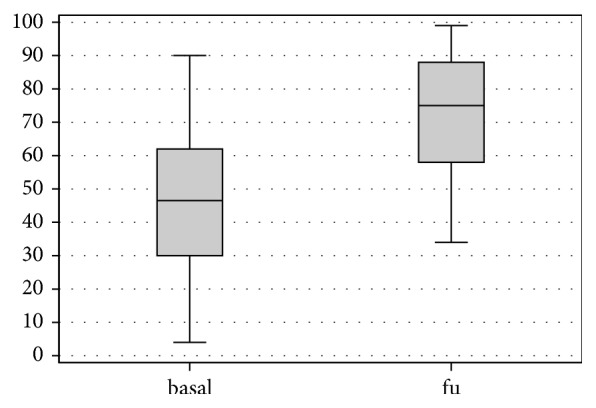
Lysholm score at basal level and at final follow-up.

**Figure 3:**
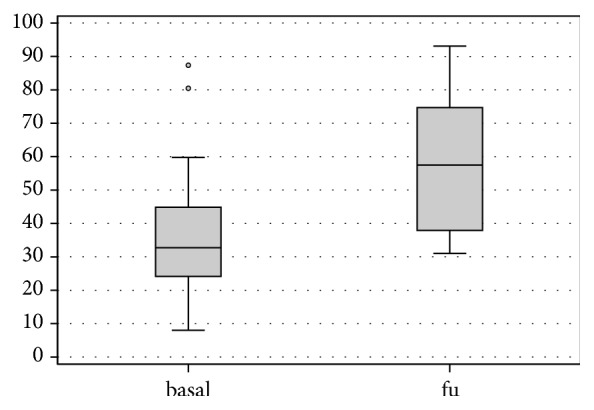
IKDC subjective outcome at basal level and at final follow-up.

**Figure 4:**
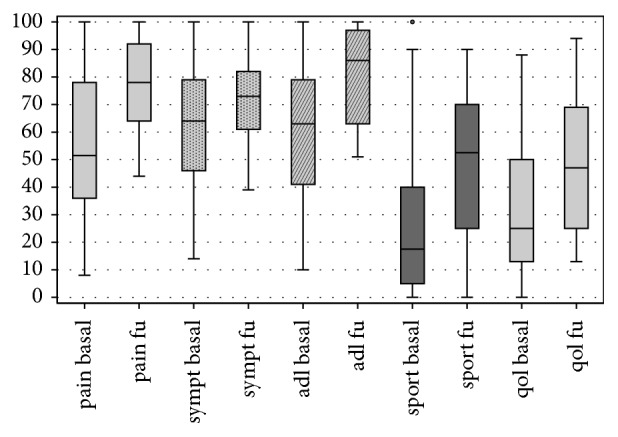
KOOS subscales at basal level and at final follow-up.

At follow-up VAS score showed a marked reduction in pain, decreasing from a mean of 67.5 ± 30.3 points preoperatively to mean 36.4 ± 25 points at the follow-up (p < .0005) (Figure 1). Lysholm Knee mean score increased from 44.1 ± 23.2 preoperatively to 73.4 ± 18.5 postoperatively (p < .0005) (Figure 2). IKDC subjective score showed an improvement in the mean scores, from 36.2 ± 20.5 to 57.0 ± 18.2 (p < .0005) (Figure 3), and KOOS score as well showed a significant improvement in all subscales: KOOS Pain from 56.3 ± 27.4 to 78.0 ± 17.0 (p < .0005), KOOS Symptoms from 61.9 ± 24.3 to 72.5 ± 15.6 (p = .006), KOOS ADL from 60.6 ± 25.0 to 81.7 ± 17.9 (p < .0005), KOOS Sport from 27.5 ± 28.6 to 50.8 ± 28.5 (p < .0005), and KOOS QOL from 32.3 ± 25.9 to 47.2 ± 22.8 (p = .012) (Figure 4). A lower and not significant improvement was shown for Tegner score, passing from 3.8 ± 1.7 to 4.4 ± 1.3 at follow-up (p = .088), without reaching the preinjury level of 6.2 ± 2.0 (p < .0005).

Previous surgical procedures were found to be significantly correlated with a worse outcome in KOOS pain (p = .025), KOOS sport (p = .036), KOOS QOL (p = .030), and IKDC (p = .038). No significant statistical correlation was found between clinical outcomes and patients sex, age, BMI, preoperative pain duration, smoking, sport activity, lesion location, and combined surgery.

Complication rate of this cases series was 11%, being represented by 1 case of scaffold resorption and 2 cases of joint stiffness (1 CFM and 1 trochlea); the latter patients underwent a second surgery for arthroscopic arthrolysis. No patient failed according to the surgical definition, while considering clinical failures only 69.2% of patients reached a clinical significant improvement evaluated with the IKDC subjective score and 30.8% of patients did not reach a significant improvement and were thus considered as clinical failure.

Finally, 19 knees were evaluated with high resolution MRI at follow-up. The MOCART evaluation showed a complete filling of the cartilage area in 63.2% of the lesions, complete integration of the graft in 47.4% of cases, intact repair tissue surface in 31.6% of the cases, homogeneous structure of the repair tissue in 42.1% of cases, and iso-intense graft signal intensity score with the adjacent native cartilage in 64.8% of the cases in both dual T2-FSE and 3D-GE-FS sequences. Moreover, the subchondral bone appearance was considered normal in 42.1%, whereas the lamina was not intact in all the cases. Finally, adhesions and effusion were shown in 26.3% and 57.9% of the cases, respectively. The mean total MOCART score was 65.0 ± 16.4. No correlation was found in this series between MOCART variables and the clinical findings.

## 4. Discussion

The most important finding of the present study is that the treatment with an osteochondral biomimetic scaffold in patients affected by EOA provides symptoms relief and function improvement at mid-term follow-up.

The clinical utility of osteochondral scaffold implantation has been previously established in a setting of nonosteoarthritic joints with focal chondral defects [8–13]. Evidence has been provided also on its use for more complex cases which entail a progressive degenerative joint environment, like tibial plateau fractures [14], and spontaneous osteonecrosis of the knee [15]. Nevertheless, there are still few evidences about the possibility to treat with success an osteochondral lesion in an already degenerated joint. Other regenerative treatments, like autologous chondrocyte implantation and matrix-assisted autologous chondrocyte transplantation, have shown less satisfactory results when applied to degenerative lesions or OA, with a high rate of failures at mid-term follow-up [3, 24]. The reason could be found in some preclinical evidences. In fact, the cytokines produced by the chondrocytes near the implant and the altered joint environment might cause dedifferentiation or apoptosis of the implanted bioengineered scaffold seeded with cells, and this might affect the results [25]. The intra-articular changes taking place in OA processes, which cause pain and effusion due to the presence of synovitis, matrix degradation, and subchondral bone changes, represent unfavorable conditions for tissue regeneration, as supported by some preclinical studies. In an animal model on goats, Saris et al. [26] showed a negative influence of a disturbed intra-articular environment on the cartilage formation, with decrease of histological, biochemical, and macroscopic parameters after tissue engineering. Similarly, Ozsoy et al. [27] showed in an experimental osteochondral defect model in rabbits a poorer outcome related to the disturbed homeostasis and the negative effects in chronic degenerative stages. Moreover, Rodrigo et al. [28] showed that the synovial fluid from the knees of patients with chronic cartilage lesions may exert an inhibitory effect, causing a negative healing environment which may impair chondrogenesis. Furthermore, tissue engineering applied to the treatment of articular degenerative lesions presents some additional problems: healthy tissues are key to provide stable sides for the implant, whereas in a degenerative process the surrounding areas may be involved, thus limiting the stability and integration of the graft.

On the other hand, some authors showed that regenerative procedures may still produce satisfactory results also in joints affected by degenerative changes. Hollander et al. [29] observed tissue regeneration even for implants in OA joints, and laboratory studies confirmed the potential usefulness of regenerative procedures in joints with degenerative lesions, even when the OA process has already started [30, 31], thus suggesting that OA does not completely inhibit the regeneration process, justifying a possible clinical use [30]. Nowadays, the only study reporting clinical results of an osteochondral scaffold in the EOA setting [16] reported a significant improvement at short-term follow-up in 23 patients diagnosed with EOA after failure of conservative management, with best results obtained for patients younger than 40 years. However, those age-related conclusions have to be considered with caution, since it has been proven that the influence of age could be related only on a score bias, as shown by a recent study where score standardization led to this difference to become not significant [32], opening to the possibility to address such osteochondral lesions also in older patients.

The cohort of 26 patients analyzed in the current study demonstrated a significant improvement between pre- and postoperative score evaluated with VAS, IKDC, KOOS, and Lysholm scores, proving a satisfactory subjective outcome in patients regardless of age. The Tegner score showed instead a not significant improvement. This could be partially explained considering that the study population suffered from a chronic condition that led to surgery after several years, in which the activity level was already lower than presymptoms levels; moreover, as the time passed, the same patients would physiologically perform at a lower intensity simply due to aging, therefore providing a worst outcome. Data gathering about complications and adverse effects was wide and comprehensive, both in the perioperative timeframe and at the follow-up trough analysis of the medical records. A complication rate of about 11% was found, represented by one case of resorption of the scaffold and two cases of joint stiffness requiring new surgery. Similar results were obtained by other study groups [33, 34]. No surgical failures were recorded during study follow-up, but 30% of these patients did not show a clinically significant improvement after treatment, which should be considered when considering this treatment indication for EOA patients [3].

This study described for the first time the MRI evaluation of the use of this biomimetic osteochondral scaffold in a EOA setting, showing less favorable results with respect to what was observed clinically: although imaging results did not correlate with the clinical outcome, only half of the patients showed complete graft integration and only one-third intact repair tissue surface. Moreover 26% and 58% of adhesion and effusion are higher rates compared to what was reported in the literature. To this regard the literature actually provides contrasting evidence. In a pilot study on 28 patients [33], the 2-year follow-up showed complete filling of the defect and integration of the border zone in 70% of the lesions, but subchondral lamina and bone were intact only in a minority of cases, not correlating with the good clinical results, and suggesting that MOCART score is not so reliable for the evaluation of the osteochondral unit as it is for the chondral layer alone. In another study by the same group, the total MOCART score of 45 cases was stable between 12 and 24 months of follow-up (72.9 ± 13.6 and 70.8 ± 13.2, respectively), and again no correlation was found between MOCART total score and the clinical parameters [16]. Different results have been published more recently by Christensen et al. [34]; using specific acquisitions and software algorithms they reported absence of defect filling, integration, and subchondral healing/restoration, utilizing MRI but also CT evaluation, despite positive clinical outcomes. The contrast about imaging and clinical results, and the absence of correlation with clinical outcomes, could be explained in consideration of the fact that current MRI scoring systems were developed for the evaluation of the chondral layer and therefore have lower specificity and sensibility in evaluating a complex organ as the osteochondral unit. Moreover, the most specific MRI acquisitions are not standard nor of easy interpretation, nor economic or available everywhere, and this can create several difficulties in comparing results of different papers. CT imaging could be suitable for the subchondral bone but fails in the needed goal of properly addressing both cartilage and subchondral bone with the same accuracy and efficacy. While the imaging findings show the limits of this osteochondral regenerative solution, especially in the EOA setting, further studies are needed to understand the significance of this findings in terms of clinical outcome.

This study presents some limitations: first of all, the small number of patients analyzed, although being the largest survey described in the literature on EOA, limits the significance of the results, being probably the reason why no correlation was found between patients' characteristics and clinical outcomes. Secondarily, such a brief follow-up, despite being sufficient to prove a clinical improvement, is obviously not optimal to evaluate the survivorship of the implant in this kind of challenging joints, which face a high risk of prosthetic replacement. Moreover, the high number of combined procedures may further jeopardize the results of the scaffold. However, knees affected by EOA are only seldom affected by a mere cartilage issue, whereas the large majority of EOA knees are affected by complex morbidity, like axial deviation, instability, meniscal lesions, etc. Thus, the patients documented in this study reflect those found in the common clinical practice. All comorbidities have to be addressed to increase the possibility of a positive outcome. Such combined biological and mechanical approach already demonstrated good results at short/medium follow-up in patients affected by unicompartmental OA eligible for prosthetic resurfacing [35–37]. The safety of performing multiple combined surgeries was further underlined in this study, where also patients with previous and combined surgeries achieve a marked clinical improvement with absence of short-term surgical failures or severe adverse events. While future biomaterial/technical improvement could further improve the potential of osteochondral implants, this treatment approach proved to be useful and could be considered a suitable option to address osteochondral lesions in patients affected by EOA.

## 5. Conclusion

The use of a biomimetic osteochondral scaffold in the setting of an EOA, alone or associated with other procedures, appeared to be a valid and safe option, able to provide good and stable clinical outcomes with high patient's satisfaction and low complication rate. Further studies on larger cohorts and with longer follow-up are need to better understand the potential of this scaffold and, of particular importance in the EOA setting, the efficacy in delaying prosthetic replacement.

## Data Availability

The clinical and radiological outcome data used to support the findings of this study are restricted by the Ethic Committee in order to protect patients' privacy. Data are available from the corresponding author for researchers who meet the criteria for access to confidential data.
